# Supplementary material for: Multiple sclerosis and abnormal spermatozoa: A bidirectional two-sample mendelian randomization study
Source: PLoS One. 2024 Aug 20;19(8):e0308815. doi: 10.1371/journal.pone.0308815 (PMC11335101; doi:10.1371/journal.pone.0308815)
Supplement: S1 Checklist — (DOCX) [file pone.0308815.s001.docx]

**STROBE-MR checklist of recommended items to address in reports of Mendelian randomization studies**^1^ ^2^

| **Item No.** | **Section** | **Checklist item** | **Page No.** | **Relevant text from manuscript** |
| --- | --- | --- | --- | --- |
| 1 | **TITLE and ABSTRACT** | Indicate Mendelian randomization (MR) as the study’s design in the title and/or the abstract if that is a main purpose of the study | 1 | The aim of this study was to assess the causal relationship between multiple sclerosis (MS) and sperm abnormalities, which was analysed using Mendelian randomisation (MR). |
|  | **INTRODUCTION** |  |  |  |
| 2 | **Background** | Explain the scientific background and rationale for the reported study. What is the exposure? Is a potential causal relationship between exposure and outcome plausible? Justify why MR is a helpful method to address the study question | 2-3 | Previous observational epidemiological studies have suggested a possible association between MS and sperm abnormalities, but the results have been inconclusive. |
| 3 | **Objectives** | State specific objectives clearly, including pre-specified causal hypotheses (if any). State that MR is a method that, under specific assumptions, intends to estimate causal effects | 1 | The aim of the study was to assess the causal relationship between MS and sperm abnormalities, and the study design included pre-specified causal assumptions, emphasising that MR is a method for estimating causal effects under conditions where certain assumptions are met. |
|  | **METHODS** |  |  |  |
| 4 | **Study design and data sources** | Present key elements of the study design early in the article. Consider including a table listing sources of data for all phases of the study. For each data source contributing to the analysis, describe the following: | 4-6 | Key elements of the study design were presented, including the use of publicly available genome-wide association study datasets to obtain instrumental variables (IVs) and the confirmation of heterogeneity and multicollinearity of these IVs (e.g. by Cochran's Q test, MR-PRESSO test and leave-one-out sensitivity analysis). The use of three different MR analytic models (inverse variance weighting method, weighted median estimation algorithm and MR-Egger regression) to estimate causal effects was also mentioned. |
|  | a) | Setting: Describe the study design and the underlying population, if possible. Describe the setting, locations, and relevant dates, including periods of recruitment, exposure, follow-up, and data collection, when available. |  |  |
|  | b) | Participants: Give the eligibility criteria, and the sources and methods of selection of participants. Report the sample size, and whether any power or sample size calculations were carried out prior to the main analysis |  |  |
|  | c) | Describe measurement, quality control and selection of genetic variants |  |  |
|  | d) | For each exposure, outcome, and other relevant variables, describe methods of assessment and diagnostic criteria for diseases |  |  |
|  | e) | Provide details of ethics committee approval and participant informed consent, if relevant |  |  |
| 5 | **Assumptions** | Explicitly state the three core IV assumptions for the main analysis (relevance, independence and exclusion restriction) as well assumptions for any additional or sensitivity analysis | 4 | The key assumptions of the MR analyses (relevance, independence and exclusionary restrictions) are clearly stated and it is reported how these assumptions are assessed against additional analyses or sensitivity analyses. |
| 6 | **Statistical methods: main analysis** | Describe statistical methods and statistics used | 5-6 | The statistical methods and statistics used are described, including how genetic variation was treated in the analyses and whether adjustments were made using the same set of covariates in the two-sample MR. |
|  | a) | Describe how quantitative variables were handled in the analyses (i.e., scale, units, model) |  |  |
|  | b) | Describe how genetic variants were handled in the analyses and, if applicable, how their weights were selected |  |  |
|  | c) | Describe the MR estimator (e.g. two-stage least squares, Wald ratio) and related statistics. Detail the included covariates and, in case of two-sample MR, whether the same covariate set was used for adjustment in the two samples |  |  |
|  | d) | Explain how missing data were addressed |  |  |
|  | e) | If applicable, indicate how multiple testing was addressed |  |  |
| 7 | **Assessment of assumptions** | Describe any methods or prior knowledge used to assess the assumptions or justify their validity | 5 | Methods used to assess or demonstrate the validity of the hypothesis, e.g. through sensitivity analyses, culling of aberrant SNPs, etc., are reported. |
| 8 | **Sensitivity analyses and additional analyses** | Describe any sensitivity analyses or additional analyses performed (e.g. comparison of effect estimates from different approaches, independent replication, bias analytic techniques, validation of instruments, simulations) | 6 | Various sensitivity analyses and additional analyses performed, including leave-one-out analyses and other testing tools, are reported. |
| 9 | **Software and pre-registration** |  | 6 | The TwoSampleMR package in R software (version 4.2.0) was used. |
|  | a) | Name statistical software and package(s), including version and settings used |  |  |
|  | b) | State whether the study protocol and details were pre-registered (as well as when and where) |  |  |
|  | **RESULTS** |  |  |  |
| 10 | **Descriptive data** |  | 7 | It was mentioned that 134 SNPs not associated with MS were screened to ensure they were not associated with potential confounders such as smoking and alcohol consumption. 124 of these SNPs were from the sperm abnormality GWAS data source. |
|  | a) | Report the numbers of individuals at each stage of included studies and reasons for exclusion. Consider use of a flow diagram |  |  |
|  | b) | Report summary statistics for phenotypic exposure(s), outcome(s), and other relevant variables (e.g. means, SDs, proportions) |  |  |
|  | c) | If the data sources include meta-analyses of previous studies, provide the assessments of heterogeneity across these studies |  |  |
|  | d) | For two-sample MR:  i.  Provide justification of the similarity of the genetic variant-exposure associations between the exposure and outcome samples  ii.  Provide information on the number of individuals who overlap between the exposure and outcome studies |  |  |
| 11 | **Main results** |  | 7-8 | Associations between genetic variants and exposure (MS) and between genetic variants and outcome (sperm abnormalities) were reported, giving the ratio of MR-estimated causal effects between exposure and outcome (OR 1.090) and their confidence intervals (95% CI [1.017-1.168]), suggesting a significant causal relationship between MS and sperm abnormalities. |
|  | a) | Report the associations between genetic variant and exposure, and between genetic variant and outcome, preferably on an interpretable scale |  |  |
|  | b) | Report MR estimates of the relationship between exposure and outcome, and the measures of uncertainty from the MR analysis, on an interpretable scale, such as odds ratio or relative risk per SD difference |  |  |
|  | c) | If relevant, consider translating estimates of relative risk into absolute risk for a meaningful time period |  |  |
|  | d) | Consider plots to visualize results (e.g. forest plot, scatterplot of associations between genetic variants and outcome versus between genetic variants and exposure) |  |  |
| 12 | **Assessment of assumptions** |  | 7-8 | Validation of key MR hypotheses is reported, including detection of heterogeneity in associations between genetic variants and exposures and outcomes, as well as possible directional confounding. |
|  | a) | Report the assessment of the validity of the assumptions |  |  |
|  | b) | Report any additional statistics (e.g., assessments of heterogeneity across genetic variants, such as *I^2^*, Q statistic or E-value) |  |  |
| 13 | **Sensitivity analyses and additional analyses** |  | 7 | Consistency comparisons were performed using multiple MR methods (e.g. IVW, WME and MER) and leave-one-out analyses were performed to assess the impact of individual SNPs on the results. In addition, potential outliers were detected and removed by MR-PRESSO. |
|  | a) | Report any sensitivity analyses to assess the robustness of the main results to violations of the assumptions |  |  |
|  | b) | Report results from other sensitivity analyses or additional analyses |  |  |
|  | c) | Report any assessment of direction of causal relationship (e.g., bidirectional MR) |  |  |
|  | d) | When relevant, report and compare with estimates from non-MR analyses |  |  |
|  | e) | Consider additional plots to visualize results (e.g., leave-one-out analyses) |  |  |
|  | **DISCUSSION** |  |  |  |
| 14 | **Key results** | Summarize key results with reference to study objectives | 7 | A significant causal association was found between MS and abnormal spermatozoa (OR 1.090, 95% CI [1.017-1.168]), while no significant association was found in reverse causality (OR 1.010, 95% CI [0.940-1.084]). |
| 15 | **Limitations** | Discuss limitations of the study, taking into account the validity of the IV assumptions, other sources of potential bias, and imprecision. Discuss both direction and magnitude of any potential bias and any efforts to address them | 10-11 | Limitations of the study are discussed, including the validation of instrumental variable assumptions in MR analyses, potential sources of heterogeneity and bias, and responses to these limitations, such as the use of sensitivity analyses and controlling for the quality of genetic variation. |
| 16 | **Interpretation** |  | 9-11 | Based on the results of the study, which should be interpreted with caution given its limitations, it has been shown that there appears to be a causal relationship between MS and abnormal sperm, which contributes to the understanding of male infertility and fertility risk in patients with MS. |
|  | a) | Meaning: Give a cautious overall interpretation of results in the context of their limitations and in comparison with other studies |  |  |
|  | b) | Mechanism: Discuss underlying biological mechanisms that could drive a potential causal relationship between the investigated exposure and the outcome, and whether the gene-environment equivalence assumption is reasonable. Use causal language carefully, clarifying that IV estimates may provide causal effects only under certain assumptions |  |  |
|  | c) | Clinical relevance: Discuss whether the results have clinical or public policy relevance, and to what extent they inform effect sizes of possible interventions |  |  |
| 17 | **Generalizability** | Discuss the generalizability of the study results (a) to other populations, (b) across other exposure periods/timings, and (c) across other levels of exposure | 11 | Discuss the generalisability of the findings to the population |
|  | **OTHER INFORMATION** |  |  |  |
| 18 | **Funding** | Describe sources of funding and the role of funders in the present study and, if applicable, sources of funding for the databases and original study or studies on which the present study is based | 13 | This study was not funded by any project. |
| 19 | **Data and data sharing** | Provide the data used to perform all analyses or report where and how the data can be accessed, and reference these sources in the article. Provide the statistical code needed to reproduce the results in the article, or report whether the code is publicly accessible and if so, where | 4 | Data analysis is based on publicly available GWAS summary data from the Ieu Open GWAS Project database. |
| 20 | **Conflicts of Interest** | All authors should declare all potential conflicts of interest | 13 | The authors have nothing to disclose. |

This checklist is copyrighted by the Equator Network under the Creative Commons Attribution 3.0 Unported (CC BY 3.0) license.

1. Skrivankova VW, Richmond RC, Woolf BAR, Yarmolinsky J, Davies NM, Swanson SA, et al. Strengthening the Reporting of Observational Studies in Epidemiology using Mendelian Randomization (STROBE-MR) Statement. JAMA. 2021;under review.

2. Skrivankova VW, Richmond RC, Woolf BAR, Davies NM, Swanson SA, VanderWeele TJ, et al. Strengthening the Reporting of Observational Studies in Epidemiology using Mendelian Randomisation (STROBE-MR): Explanation and Elaboration. BMJ. 2021;375:n2233.
