# Supplementary material for: Multiple sclerosis and abnormal spermatozoa: A bidirectional two-sample mendelian randomization study
Source: PLoS One. 2024 Aug 20;19(8):e0308815. doi: 10.1371/journal.pone.0308815 (PMC11335101; doi:10.1371/journal.pone.0308815)
Supplement: Attachment — Submitted filename: Response to Reviewers.docx [file pone.0308815.s004.docx]

**Response to Reviewers**

Dear David Chau and reviewers:

Thank you for your letter and the reviewers’ comments on our manuscript entitled " Multiple Sclerosis and abnormal spermatozoa: A Bidirectional Two-Sample Mendelian randomization study" (ID：PONE-D-24-10655). Those comments are very helpful for revising and improving our paper, as well as the important guiding significance to our researches. We have studied the comments carefully and made corrections which we hope meet with approval. The main corrections are in the manuscript and the responses to the editor’s and reviewers’ comments are as follows (the replies are highlighted in blue ).

Replies to general comments from the editor:

1. *Please ensure that your manuscript meets PLOS ONE's style requirements, including those for file naming.*

Thanks for your careful checks. Changes have been made to our manuscript to comply with PLOS ONE journal formatting requirements.

2. *We noticed you have some minor occurrence of overlapping text with the following previous publication(s), which needs to be addressed:*

*https://www.frontiersin.org/journals/immunology/articles/10.3389/fimmu.2023.1126457/full*

*https://wjmh.org/DOIx.php?id=10.5534/wjmh.220109*

*In your revision ensure you cite all your sources (including your own works), and quote or rephrase any duplicated text outside the methods section. Further consideration is dependent on these concerns being addressed.*

We extend our sincerest apologies for the inappropriate language used in the manuscript. In our resubmitted manuscript, the typo is revised. Thanks for your correction.

3. *Please include captions for your Supporting Information files at the end of your manuscript, and update any in-text citations to match accordingly. Please see our Supporting Information guidelines for more information:* [*http://journals.plos.org/plosone/s/supporting-information*](http://journals.plos.org/plosone/s/supporting-information)*.*

Thanks for your careful checks. We have included appropriate headings for the additional information and updated their citations in the manuscript.

4. *Please review your reference list to ensure that it is complete and correct. If you have cited papers that have been retracted, please include the rationale for doing so in the manuscript text, or remove these references and replace them with relevant current references. Any changes to the reference list should be mentioned in the rebuttal letter that accompanies your revised manuscript. If you need to cite a retracted article, indicate the article’s retracted status in the References list and also include a citation and full reference for the retraction notice.*

We feel sorry for our carelessness. The list of reference information has been completed.

Responds to the reviewer’s comments:

Reviewer #1:

1. *The abstract could be improved in several ways, as it could benefit from more specific information about the MR methods used, such as the inverse-variance weighted (IVW) model, the weighted median estimator (WME) model, and the MR-Egger regression (MER) model. Furthermore, the presentation of the results in the abstract could be more concise and focused on the key findings. For example, the abstract could mention the odds ratio and confidence interval for the causal relationship between MS and abnormal sperm.*

Thank you very much for your careful review, which helped us a lot with the article. Following your suggestions, we have revised the methods and conclusions in the abstract section to make the methods more substantial and the conclusions more concise. Please see page 2 of the revised manuscript, lines 28–39, and page 3, lines 40–41.

2. *The introduction could benefit from a more detailed discussion of the neural substrates involved in the relationship between MS and abnormal sperm. I believe that this would provide a stronger foundation for the study and help situate it within the broader context of neuroimmunology and male reproductive health [1-4].*

Thank you for your constructive comments on our article, we have introduced relevant literature into our article. Please see page 5 of the revised manuscript, lines 85–92.

3. *The study mainly included participants of European descent, which may lead to biased estimates and limit the generalizability of the findings. It would be beneficial to include a more diverse population to enhance the external validity of the results.*

Thank you for your suggestion, this is indeed a shortcoming of this study for which we apologise. As the majority of data in the publicly available database is of European ethnicity, data of other ethnicities could not be found, and this shortcoming is also described in the discussion section of the article. We will continue to refine the data content of this study if other ethnicities are subsequently found to be available in the database.

4. *The GWAS summary data used did not report specific characteristics of abnormal spermatozoa, such as concentration, vitality, and morphology. This lack of detailed information could impact the accuracy of the results and hinder the ability to control for potential confounders effectively.*

Thank you for your careful review and we apologise for this, as with the above response, as the current publicly available database does not include specific breakdowns of specific sperm concentration, viability and morphology and we will continue to refine the data content of this study if data on specific sperm parameter breakdowns are subsequently found to be available in the database.

5. *While the study employed Mendelian randomization (MR) analysis, which is a robust method, there could be further exploration of potential confounding factors that were not addressed in the analysis. Additionally, the study could benefit from a more detailed explanation of the selection criteria for instrumental variables (IVs) to ensure the validity of the MR analysis.*

Thank you for your valuable comments on the article, following your suggestions we have further analyzed confounders in Mendelian randomization studies and explained the choice of instrumental variables in more detail. Please see page 8 of the revised manuscript, lines 151-171 and page 9, lines 174-216.

6. *The study found a causal relationship between MS and abnormal spermatozoa, but it is essential to acknowledge the limitations of MR analysis, such as assumptions of no horizontal pleiotropy. Providing a more nuanced discussion on the implications of the findings and potential alternative explanations would strengthen the paper.*

We feel great thanks for your professional review work on our article. We discuss the results and possible alternative explanations in more detail. Please see page 12 of the revised manuscript, lines 229-248.

7. *Please cite more references. An original study like this typically cites more than 60-70 references.*

Thanks to your suggestion, we have added references cited in the discussion section to explore various aspects of the possible causes of abnormal spermatozoa in MS. Please see page 15 of the revised manuscript, lines 285-323 and page 18, lines 374-356.

Reviewer #2:

*The paper is interesting and well written. The authors conducted a Mendelian randomization (MR) analysis to evaluate the causal relationship between multiple sclerosis (MS) and abnormal sperm.Three different models of MR analysis were performed: the inverse-variance weighted (IVW) model, the weighted median estimator (WME) model, and the MR-Egger regression (MER) model. The methodology is coerent and adequate to the endpoints of the study. The statistical analysis is adequate and well defined. The results are clear and discussion is coerent with the results and the endpoints of the study. The paper may be acceptable for publication.Statistical analysis Minor english editing.*

Thank you for your suggestions, we have revised the article where the expression was inappropriate and once again we are very grateful for your professional review work.

According to the reviewer’s comments, we have revised the manuscript extensively. If there are any other modifications we could make, we would like very much to modify them and we really appreciate your help. “PLOS ONE” is a journal of great popularity and prestige. We hope that our manuscript could be considered for publication in your journal. Thank you very much for your help.

Kind regards

Bo Li

Corresponding author :Jianwen Quan

E-mail: yczxyyqjw@163.com(JWQ)
