# Supplementary material for: Multiple sclerosis and abnormal spermatozoa: A bidirectional two-sample mendelian randomization study
Source: PLoS One. 2024 Aug 20;19(8):e0308815. doi: 10.1371/journal.pone.0308815 (PMC11335101; doi:10.1371/journal.pone.0308815)
Supplement: Attachment — Submitted filename: Response to Reviewer.docx [file pone.0308815.s005.docx]

**Response to Reviewers**

Dear David Chau and reviewers:

Thank you again for your letter and the reviewers' comments on our manuscript entitled "Multiple Sclerosis and Sperm Abnormalities: A Two-Way Two-Sample Mendelian Randomised Study" (ID: PONE-D-24-10655).These comments help to improve the scientific rigour of our article. Based on your suggestions and requests, we have made corrections to the revised manuscript. All the revised parts are marked in red in the revised manuscript, which we would like to submit for your kind consideration.

Replies to general comments from the editor:

1. *Albeit a large number of SNPs analysed, please ensure to use a relatively high* *genome wide significance threshold ie p < 10^-8 in order to produce an enough significant SNPs.*

Thank you very much for your valuable comments. Increasing the genome-wide significance threshold will reduce the number of SNPs, which will affect the analysis of the data. And there have been several similar studies using a genome-wide significance threshold of p < 10^-6 (PMID: 36077729[1], PMID: 33243239[2], PMID: 32186652[3]). In subsequent studies, we will look for more data on multiple sclerosis and use higher thresholds for data analysis to validate our analysis.

1. Garcia-Etxebarria K, Etxart A, Barrero M, Nafria B, Segues Merino NM, Romero-Garmendia I, Franke A, D'Amato M, Bujanda L. Performance of the Use of Genetic Information to Assess the Risk of Colorectal Cancer in the Basque Population. Cancers (Basel). 2022 Aug 29;14(17):4193. doi: 10.3390/cancers14174193 . PMID: 36077729; PMCID: PMC9454881. IF: 4.5 Q1.

2. Kwok MK, Kawachi I, Rehkopf D, Schooling CM. The role of cortisol in ischemic heart disease, ischemic stroke, type 2 diabetes, and cardiovascular disease risk factors: a bi-directional Mendelian randomization study. BMC Med. 2020 Nov 27;18(1):363. doi: 10.1186/s12916-020-01831-3IF: 7.0 Q1 . PMID: 33243239IF: 7.0 Q1 ; PMCID: PMC7694946.IF: 7.0 Q1 .

3. Chen HY, Cairns BJ, Small AM, Burr HA, Ambikkumar A, Martinsson A, Thériault S, Munter HM, Steffen B, Zhang R, Levinson RT, Shaffer CM, Rong J, Sonestedt E, Dufresne L, Ljungberg J, Näslund U, Johansson B, Ranatunga DK, Whitmer RA, Budoff MJ, Nguyen A, Vasan RS, Larson MG, Harris WS, Damrauer SM, Stark KD, Boekholdt SM, Wareham NJ, Pibarot P, Arsenault BJ, Mathieu P, Gudnason V, O'Donnell CJ, Rotter JI, Tsai MY, Post WS, Clarke R, Söderberg S, Bossé Y, Wells QS, Smith JG, Rader DJ, Lathrop M, Engert JC, Thanassoulis G. Association of FADS1/2 Locus Variants and Polyunsaturated Fatty Acids With Aortic Stenosis. JAMA Cardiol. 2020 Jun 1;5(6):694-702. doi: 10.1001/jamacardio.2020.0246. PMID: 32186652; PMCID: PMC7081150.IF： 14.8 Q1

2. *To include details on multiple testing correction to justify for an accurate data analysis.*

In this study, a variety of MR analysis methods (including Inverse Variance Weighted, MR Egger, Weighted Median) were comprehensively applied, with the Inverse Variance Weighted (IVW)-based method demonstrating outstanding statistical efficacy and significant superiority compared to other MR analysis strategies. To ensure the robustness of the IVW estimation conclusions, we also performed sensitivity analyses complemented by other MR analysis models.

The Inverse Variance Weighted (IVW) heterogeneity indicator (based on Cochran Q test with p-value less than 0.05) is used to indicate the possible presence of horizontal multidirectionality, and in the present study the Cochran Q test showed that there was no heterogeneity in p-values for both MR-Egger (p=0.385) and IVW (p=0.383) analyses. The intercept values obtained from the MR-Egger regression analyses were used as an indicator of the presence of directional multidirectionality, with p-values less than 0.05 considered evidence of directional multidirectionality, and analysis of the data showed that the intercept values for the MR-Egger regression were not significantly different (intercept=0.001; p=0. 890). Using leave-one-out cross-validation to assess the contribution of each single nucleotide polymorphism (SNP) to the overall Mendelian random (MR) effect estimate and its potential impact on bias, no single SNP was found to have a significant impact on the overall results in the sensitivity analyses where individual SNPs were excluded one at a time (Figure 2).

In addition, the present study followed the current practice of most similar studies by maintaining consistency in the direction of effect sizes (beta values) (IVW: 1.090, MR Egger: 1.083, weighted median 1.070) in the application of all MR analysis techniques, a move that increased the reliability of the experimental results. These are described in detail in the data analysis section of the paper.

3. *it is necessary to use literature or a tool such as phenoscanner to validate whether the identified SNPs are associated with any other outcome or exposure.*

To assess potential confounding effects and ensure accurate interpretation of genetic variants, this study used an online analysis resource, the Single Nucleotide Polymorphism Annotator (https://snipa.helmholtz-muenchen.de/snipa3/), to perform multiplicity analyses of SNPs.

4. *interpretation and conclusions drawn must be supported by a robust methodology in order to fulfill our publication criteria 3 (*[*https://journals.plos.org/plosone/s/criteria-for-publication*](https://journals.plos.org/plosone/s/criteria-for-publication)*).*

This study used Mendelian randomisation to analyse the data. Mendelian randomisation analysis is a genetics-based statistical tool that uses naturally occurring genetic variation to mimic a randomised trial to investigate causal effects between exposures and health outcomes, overcoming the limitations of observational studies. By using genetic variation as an instrumental variable and analysing its effect on exposure and outcome to infer causality, this method mimics the rigour of a randomised controlled trial but avoids the ethical and practical difficulties of doing so in practice.

Sensitivity analyses play a key role in this framework and are used to validate the stability and reliability of the results. This includes detecting heterogeneity in the data, assessing the pleiotropic effects of genetic variants (i.e. the potential for a genetic variant to affect multiple traits), performing analyses that step down individual genetic markers, restricting analyses to the most reliable genetic variants, and checking for publication bias using funnel plots. These steps ensure that results withstand different assumptions and model variations, thereby increasing confidence in causal inferences, and are central to ensuring the scientific validity and effectiveness of Mendelian randomisation analyses.

Responds to the reviewer’s comments:

Reviewer #1:

*1.Keywords: Please include six keywords from Medical Subject Headings (MeSH) in the title and the first two sentences of the abstract.*

The context in the abstract section has been changed, thank you for your constructive comments. Please see page 2 of the revised manuscript, lines 22–29.

*2. Abstract: I recommend that the authors present the background, methods, results, and conclusion in a proportional order within 200 words without subheadings. The general background (one to two sentences), specific background (two to three sentences), and current issue addressed in this study (one sentence) should all be included in the background before moving on to the objectives. In this subsection, I would like the authors to provide background information, a problem statement, and their reasoning for breaking off. The results section ends with a phrase that places this subsection in a broader context. The conclusion should start with a single sentence that summarizes the main message, such as "Here we show." The authors should describe the potential and progress of this study in the field in the first sentence of the conclusion, followed by two to three sentences that provide a broader perspective that any scientist can comprehend.*

We'd like to thank you for these valuable comments, which have been carefully edited and summarised. Please see the abstract on page 2, lines 22-49 of the revised manuscript.

*3. Conclusion: To effectively communicate the manuscript's main message, I recommend dedicating a single paragraph, approximately 150-200 words long, to highlight the authors' extensive and thorough considerations as esteemed experts in their respective fields. This approach would be advantageous because it emphasizes the importance of their efforts to explain the theoretical implications and practical applications of their findings. It is also critical to discuss potential areas for future research, as well as theoretical and methodological aspects that require further development, in order to fully comprehend the significance of this line of research.*

Thank you for your expert advice on our article. The conclusion section has been carefully amended as requested. Please see the conclusions section on lines 383-394 on page 20 of the revised manuscript.

In response to the valuable feedback from the reviewers, we have meticulously revised our manuscript with the aim of aligning it with the esteemed publication standards of "PLOS ONE", a journal renowned for its high prestige and wide readership. We sincerely hope that this refined submission will merit your consideration for publication in your esteemed journal. We sincerely appreciate your continued support and guidance throughout the review process.

Kind regards

Bo Li

Corresponding author :Jianwen Quan

E-mail: yczxyyqjw@163.com(JWQ)
